# Glial and synaptic biomarkers as early indicators of mild cognitive decline

**DOI:** 10.1002/alz.088938

**Published:** 2025-01-03

**Authors:** Agnieszka Kulczynska‐Przybik, Maciej Dulewicz, Henrik Zetterberg, Jörg Hanrieder, Kaj Blennow, Jan Mroczko, Daria Krawczuk, Julia Doroszkiewicz, Renata Borawska, Agnieszka Slowik, Barbara Mroczko

**Affiliations:** ^1^ Department of Neurodegeneration Diagnostics, Medical University of Bialystok, Bialystok Poland; ^2^ Department of Psychiatry and Neurochemistry, Institute of Neuroscience and Physiology, The Sahlgrenska Academy at the University of Gothenburg, Gothenburg, Gothenburg Sweden; ^3^ Department of Psychiatry and Neurochemistry, Institute of Neuroscience and Physiology, The Sahlgrenska Academy at the University of Gothenburg, Gothenburg Sweden; ^4^ Institute of Neuroscience and Physiology Sahlgrenska Academy at the University of Gothenburg, Gothenburg Sweden; ^5^ Department of Neurology, Jagiellonian University, Krakow Poland

## Abstract

**Background:**

Cognitive disorders are a growing cause of morbidity and mortality worldwide. Diagnostic approaches to improve early diagnosis of cognitive disorders are constantly being sought. The pathogenesis of cognitive impairment is multifactorial and complex. It is believed that microglial activation and synaptic disturbance are crucial mechanisms leading to disease progression thus study of the interrelationships of the proteins involved in these processes appears to be important. Chemokine CX3CL1 seems to play a pivotal role in the central nervous system and it is involved in microglia‐neuron communication, neuronal survival, synaptic plasticity, as well as neuronal excitability. Findings from preclinical studies on AD models have reported the crucial role of CX3CL1 in microglial activation, regulation of plaque load, and cognition. Neurogranin is considered, as a biomarker of early synaptic dysfunction in AD. Additionally, it may serve as a marker to predict disease progression. Therefore, we aimed to investigate and compare CX3CL1 and neurogranin (Ng) levels in CSF patients with mild cognitive decline (MCI) and cognitively normal subjects from the control group. We also assessed an association between CSF concentrations of CX3CL1, Ng, and neurochemical dementia biomarkers.

**Method:**

The concentrations of CX3CL1, neurogranin as well as neurochemical dementia biomarkers, including amyloid beta 1‐42 (Aß‐42), amyloid beta 1‐40, Tau, as well as pTau181 were measured in cerebrospinal fluid patients with mild cognitive impairment and individuals without cognitive decline by multiplexing and enzyme‐linked immunosorbent techniques.

**Result:**

Significantly higher CSF concentrations of CX3CL1 and neurogranin were found in MCI patients in comparison to subjects without cognitive decline. Furthermore, in the group of patients with MCI the CSF levels of CX3CL1 and Ng significantly correlated with Aβ‐42, and pTau181 proteins. Similarly, a significant association between neurogranin and Tau protein was observed. Additionally, a positive correlation between CSF levels of Ng and CX3XL1 was noticed.

**Conclusion:**

Our findings indicate that both proteins CX3CL1, as well as neurogranin, could be applied as complementary early biomarkers for more accurate diagnosing and stratification of patients with cognitive decline.

